# Up-regulation of platelet-activating factor synthases and its receptor in spinal cord contribute to development of neuropathic pain following peripheral nerve injury

**DOI:** 10.1186/1744-8069-8-8

**Published:** 2012-02-02

**Authors:** Masamichi Okubo, Hiroki Yamanaka, Kimiko Kobayashi, Hirosato Kanda, Yi Dai, Koichi Noguchi

**Affiliations:** 1 Department of Anatomy and Neuroscience, Hyogo College of Medicine, Nishinomiya, Hyogo 663-8501, Japan; 2 Department of Pharmacy, School of Pharmacy, Hyogo University of Health Sciences, Kobe, Hyogo 650-8530, Japan; 3 Department of Neural and Pain Sciences, University of Maryland Dental School, Baltimore, MD 21201, USA

**Keywords:** PAF, Synthase, Receptor, Microglia, Neuron, Neuropathic pain

## Abstract

**Background:**

Platelet-activating factor (PAF; 1-alkyl-2-acetyl-sn-glycero-3-phosphocholine) is a lipid mediator derived from cell membrane. It has been reported that PAF is involved in various pathological conditions, such as spinal cord injury, multiple sclerosis, neuropathic pain and intrathecal administration of PAF leads to tactile allodynia. However, the expression of PAF synthases and its receptor in the spinal cord following peripheral nerve injury is unknown.

**Methods:**

Using the rat spared nerve injury (SNI) model, we investigated the expression of PAF synthases (LPCAT1 and 2) and PAF receptor (PAFr) mRNAs in the spinal cord. Reverse transcription polymerase chain reaction (RT-PCR) and double-labeling analysis of *in situ *hybridization histochemistry (ISHH) with immunohistochemistry (IHC) were employed for the analyses. Pain behaviors were also examined with PAFr antagonist (WEB2086).

**Results:**

RT-PCR showed that LPCAT2 mRNA was increased in the ipsilateral spinal cord after injury, but not LPCAT1 mRNA. Double-labeling of ISHH with IHC revealed that LPCAT1 and 2 mRNAs were constitutively expressed by a subset of neurons, and LPCAT2 mRNA was increased in spinal microglia after nerve injury. RT-PCR showed that PAFr mRNA was dramatically increased in the ipsilateral spinal cord after nerve injury. Double-labeling analysis of ISHH with IHC revealed that after injury PAFr mRNA was predominantly colocalized with microglia in the spinal cord. Continuous intrathecal administration of the PAFr antagonist suppressed mechanical allodynia following peripheral nerve injury. Delayed administration of a PAFr antagonist did not reverse the mechanical allodynia.

**Conclusions:**

Our data show the histological localization of PAF synthases and its receptor in the spinal cord following peripheral nerve injury, and suggest that PAF/PAFr signaling in the spinal cord acts in an autocrine or paracrine manner among the activated microglia and neurons, thus contributing to development of neuropathic pain.

## Background

Peripheral nerve injury can cause neuropathic pain syndromes characterized by both spontaneous and evoked painful sensations. Although it is thought that plastic alterations in central or peripheral neuronal processing play important roles in the development of neuropathic pain [1-5], the underlying molecular mechanisms are not fully understood. Accumulated evidence shows that glial cells in the spinal cord significantly contribute to neuropathic pain [6,7] and that after peripheral nerve injury activated glial cells produce several inflammatory molecules [8]. Recently, we have reported that leukotrienes, one of the lipid mediators produced by glial cells, are involved in the development of neuropathic pain following peripheral nerve injury [9].

The platelet-activating factor (PAF; 1-alkyl-2-acetyl-sn-glycero-3-phosphocholine) is a lipid mediator derived from cell membrane and implicated in a variety of physiological and pathological conditions [10-12]. LysoPAF, a precursor of PAF, is produced from glycerophospholipid cleaved by Ca^2+^-dependent cytosolic phospholipase A2 (cPLA2). LysoPAF is converted to PAF by lysophosphatidylcholine acyltransferase 1 (LPCAT1) or acetyl-CoA:lyso-PAF acetyltransferase/lysophosphatidylcholine acyltransferase 2 (LPCAT2) enzymatically [13,14]. PAF binds the PAF receptor (PAFr) that coupled to G proteins Gi, Gq, and G12/13. Activation of PAFr results in the mobilization of intracellular Ca^2+^, inhibition of cyclic AMP formation and the activation of mitogen-activated protein kinases. Thus, it appears that PAFr can induce a variety of intracellular signaling pathways that evoke wide range of biological functions [10,15,16].

In the nervous system, PAF is involved in pathological conditions, such as ischemia-reperfusion injury, spinal cord injury and multiple sclerosis [17-19]. Several reports have suggested a role of the PAF/PAFr system in modulating pain signaling in the peripheral nervous system. PAF is involved in ultraviolet B irradiation-induced hyperalgesia in the rat hindpaw [20] and intraplantar injection of PAF induced hypersensitivity in response to noxious stimuli [21,22]. Recently, Hasegawa et al. have demonstrated that dorsal root ganglion (DRG) neurons express LPCAT2 and macrophages around the DRG neurons express PAFr after peripheral nerve injury. The underlying mechanism of pain signaling induced by PAF in the peripheral nervous system is that the activation of PAFr may produce several proinflammatory cytokines after nerve injury [23]. In the central nervous system, it has also been reported that PAF is implicated in the induction of pain behaviors. Morita et al. have demonstrated that intrathecal injection of PAF produced potent tactile allodynia in mice, suggesting that PAF in the spinal cord may be a mediator of neuropathic pain following peripheral nerve injury [24,25]. The expression of PAF synthases and PAFr in the spinal cord is unknown and accumulating evidence has led us to investigate the histological evidence of PAF synthases and PAFr in spinal cord and to study whether the PAF/PAFr pathway plays a role in neuropathic pain induced by peripheral nerve injury. The purpose of present study was to examine the detailed expression pattern of PAF synthases and its receptor in the rat spinal cord after nerve injury and to confirm their roles in neuropathic pain.

## Results

### Peripheral nerve injury increases LPCAT2 mRNA in spinal microglia

**Figure 1:**
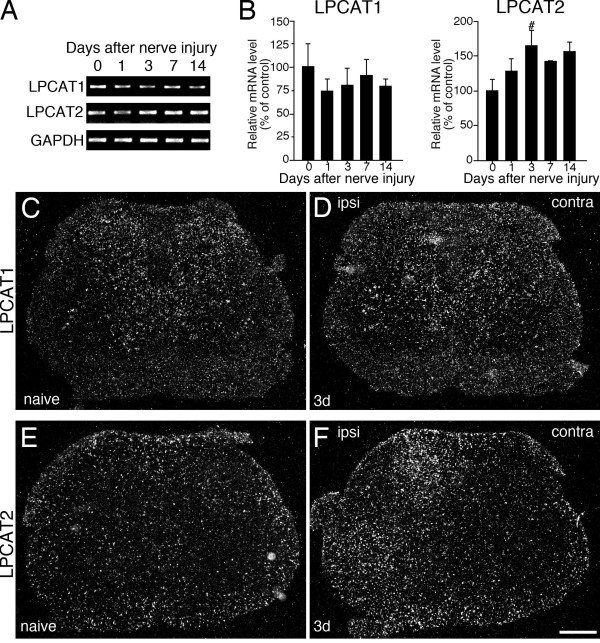
**Peripheral nerve injury (SNI) up-regulates LPCAT2 mRNA in the ipsilateral spinal cord**. (A) Gel panels show PCR products from the ipsilateral L4-L5 spinal cord taken from 0 (naive), 1, 3, 7 and 14 days after nerve injury. (B) Graphs show quantification of the relative mRNA levels of LPCAT1 and LPCAT2. LPCAT1 and LPCAT2 mRNA levels were normalized against GAPDH (n = 4, mean ± SEM, #; *p *< 0.05 compared with naive). (C-F) Darkfield images of ISHH revealed the mRNA expression of LPCAT1 (C, D) and LPCAT2 (E, F) in naive rats (C, E) and 3 days after nerve injury (D, F). Calibration bar: 500 μm.

**Figure 2:**
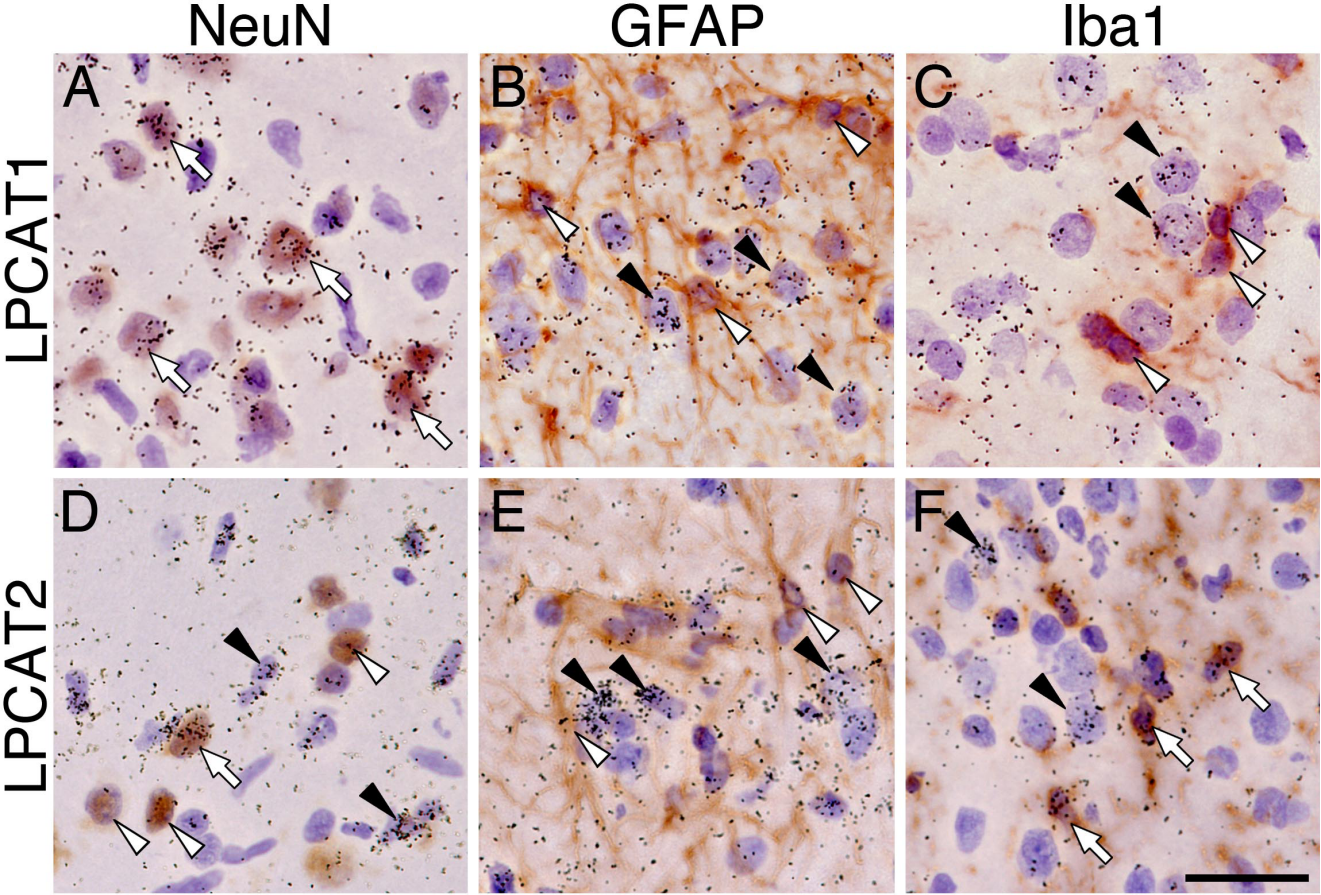
**LPCAT2 mRNA is increased by spinal microglia after SNI surgery**. Bright-field images of combined ISHH for LPCAT1 (A-C) and LPCAT2 (D-F) with IHC for NeuN (A, C), GFAP (B, E) and Iba1 (C, F) at 3 days after SNI surgery. Open arrows indicate double-labeled cells. Arrowheads indicate single-labeled cells by ISHH (aggregation of grains), and open arrowheads indicate single immunostained cells (brown staining). Calibration bar: 20 μm.

To examine whether the induction of PAF synthase mRNAs in rat spinal cord occurs after nerve injury, we first investigated reverse transcription polymerase chain reaction (RT-PCR) for LACAT1 and LPCAT2 mRNAs in L4-L5 ipsilateral spinal cord tissue that received spared nerve injury (SNI) surgery (n = 4, each time point). RT-PCR revealed that LPCAT2 was significantly increased at 3 days after nerve injury (Figure 1A, B). LPCAT1 mRNA did not change after SNI surgery (Figure 1A, B). We examined the expression pattern of LPCAT1 in the spinal cord of naive and 3 days after nerve injury using *in situ *hybridization histochemistry (ISHH) with radioisotope-labeled probes (Figure 1C, D). In the spinal cord of naive rats, we detected LPCAT1 mRNA signals throughout the spinal cord (Figure 1C). As predicted from the RT-PCR analysis, the expression of LPCAT1 mRNA was not changed after nerve injury (day 3) compared to naive rats using ISHH (Figure 1D). Next, we demonstrated the distribution of mRNA for LPCAT2 in the spinal cord after SNI surgery (Figure 1E, F). We detected signals for LPCAT2 in both white and gray matter of the naive rat spinal cord (Figure 1E) and the LPCAT2 mRNA was significantly increased at 3 days after surgery on the ipsilateral side (Figure 1F). The aggregation of grains occurred in cells with small nuclei stained by hematoxylin (data not shown). To characterize the LPCAT1 and LPCAT2 mRNA-expressing cells in the spinal cord after SNI (day 3), we performed double-labeling analysis of ISHH with immunohistochemistry (IHC) for NeuN, GFAP and Iba1 (Figure 2). We found that hybridization signals for LPCAT1 mRNA were predominantly localized in NeuN-positive cells, but not GFAP or Iba1-positive cells, thus indicating that the LPCAT1 expression was in neurons (Figure 2A-C). LPCAT2 mRNA-expressing cells were not labeled by GFAP immunoreactivity (Figure 2E). The LPCAT2 mRNA-expressing cells were double-labeled with a small number of NeuN-positive cells both in the contralateral (data not shown) and ipsilateral spinal dorsal horn after nerve injury. In contrast, LPCLT2 mRNA positive signals were heavily colocalized with Iba1 immunoreactivities in the dorsal horn ipsilateral to the injury (Figure 2F). Therefore, these data suggest that both LPCAT1 and 2 mRNAs were constitutively expressed in a small subset of neurons and LPCAT2 was predominantly increased in microglia following peripheral nerve injury.

### PAF receptor mRNA was exclusively increased in spinal microglia after peripheral nerve injury

**Figure 3:**
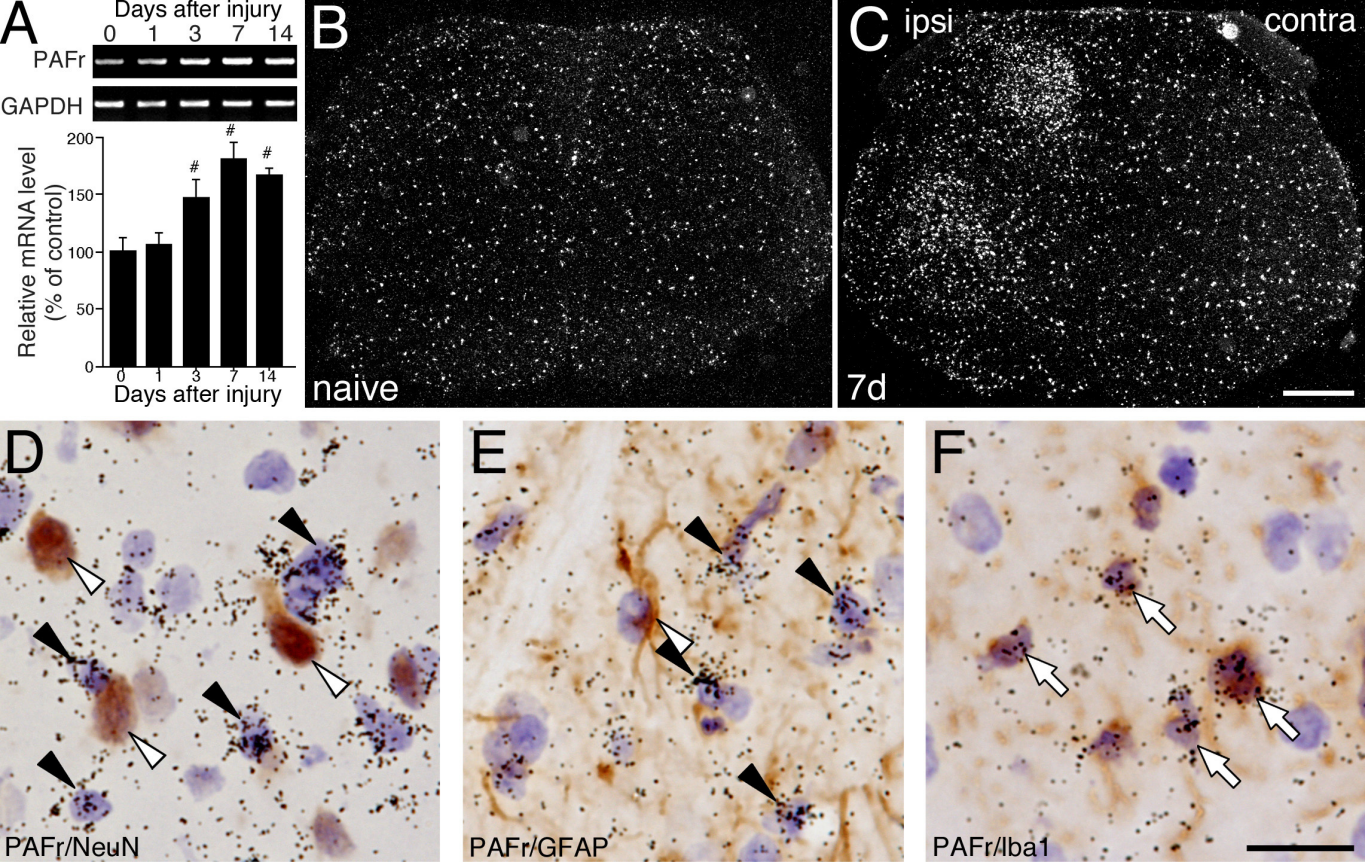
**Peripheral nerve injury enhances the expression of mRNA for PAF receptor in ipsilateral spinal microglia**. (A) Upper panels show PCR products from the ipsilateral L4-L5 spinal cord taken from 0 (naive), 1, 3, 7 and 14 days after nerve injury. Lower graphs show the statistical quantification of the relative mRNA levels of PAFr. PAFr mRNA level was normalized against GAPDH (n = 4, mean ± SEM, #; *p *< 0.05 compared with naive). (B, C) Darkfield photographs reveal the ISHH for PAFr mRNA expression in the spinal cord taken from naïve rats (B) or 7 days (C) after SNI surgery. (D-F) Characterization of SNI-induced PAFr mRNA in the spinal dorsal horn. Brightfield photographs of combined ISHH for PAFr with IHC for NeuN (D), GFAP (E) and Iba1 (F) at 7 days after injury. Open arrows indicate double-labeled cells. Arrowheads indicate single-labeled cells by ISHH (aggregation of grains), and open arrowheads indicate single immunostained cells (brown staining). Calibration bars: darkfield images; 500 μm, brightfield images; 20 μm.

The expression of PAFr mRNA in the spinal cord was also examined by RT-PCR analysis. RT-PCR revealed that mRNA of PAFr significantly increased, peaked at 7 days and continued at least for 14 days after nerve injury (Figure 3A). In order to determine the expression pattern of PAFr mRNA, we performed ISHH in the spinal cord after peripheral nerve injury using ISHH. As predicted from the RT-PCR analysis, the expression of PAFr mRNA in the spinal cord was increased in ipsilateral dorsal and ventral horn at 7 days after nerve injury compared with naive rats (Figure 3B, C). The labeled cells for PAFr mRNA contained small nuclei deeply stained by hematoxylin (data not shown). To elucidate the cell type of PAFr mRNA positive cells, we carried out the double-labeling study of ISHH with IHC (Figure 3D-F). The double-labeling analysis revealed that the induction of PAFr mRNA in the spinal cord occurred in cells labeled for Iba1 at 7 days after nerve injury (Figure 3F), not in those labeled with NeuN or GFAP (Figure 3D, E). These data suggested that PAFr mRNA was exclusively expressed in microglia in the ipsilateral spinal cord after nerve injury.

### Intrathecal administration of PAF receptor antagonist reduced mechanical allodynia induced by peripheral nerve injury

**Figure 4:**
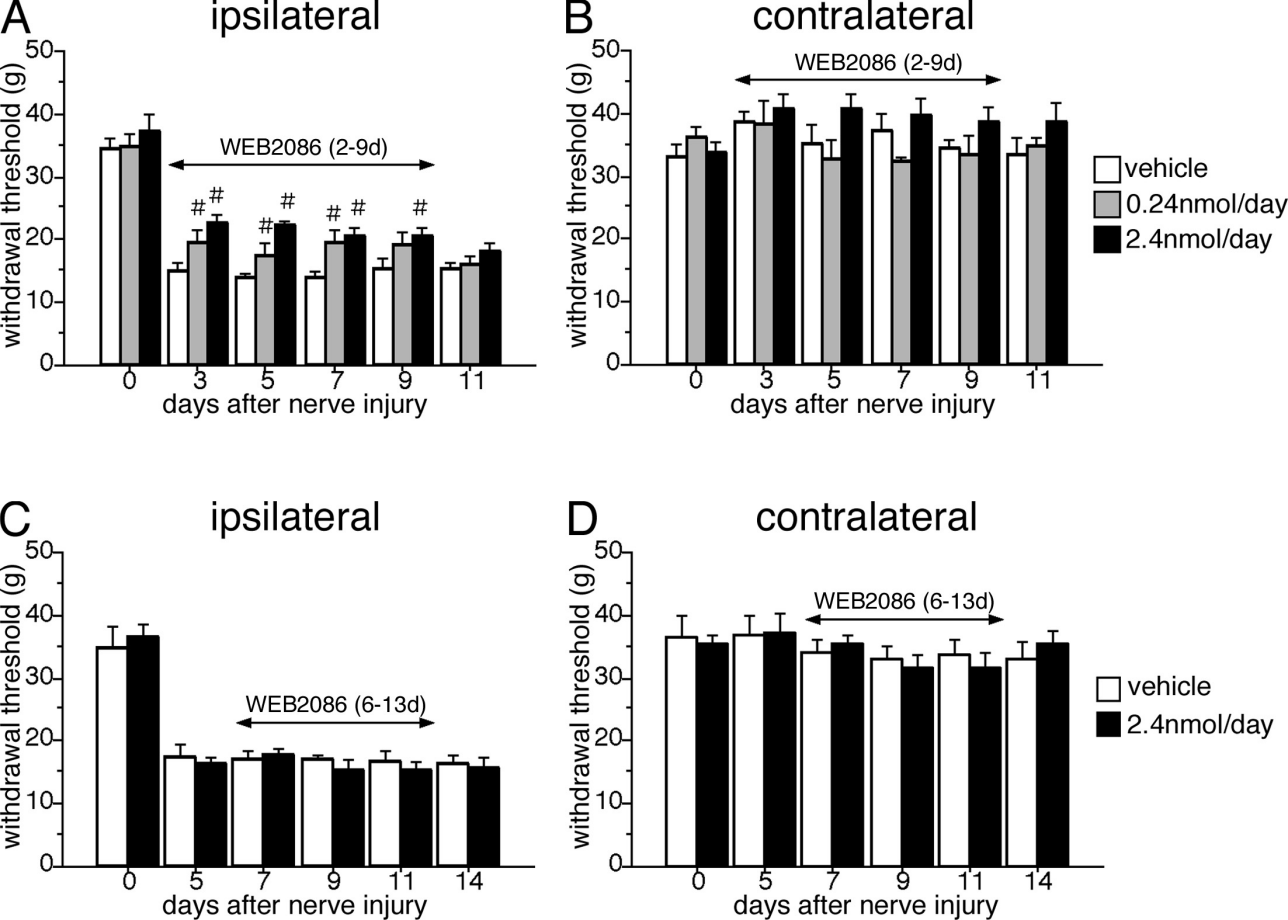
**Intrathecal administration of PAF receptor antagonist (WEB2086) significantly reduces the mechanical allodynia following peripheral nerve injury**. (A, B) The osmotic pump was set 2 days after nerve injury and drug administration continued for 7 days. (C, D) The osmotic pump was implanted 6 days after SNI surgery and worked for 7 days. (B, D) The treatment with these drugs did not change the mechanical sensitivity on the contralateral side. In all graphs, values are mean ± SEM (*n *= 6-7 in each group, # *p *< 0.05 compared with vehicle).

The presence of the mRNA of synthases and the receptor for PAF in the spinal cord after nerve injury led us to behavioral experiments to examine whether PAF has a role in neuropathic pain. The effect of an intrathecal injection of the PAFr antagonist (WEB2086) was examined using the SNI model (Figure 4). WEB2086 has been used as a specific antagonist for PAFr [24]. An osmotic pump was set and the administration of antagonist started from 2 days after SNI surgery. The nerve injury decreased the withdrawal threshold on the ipsilateral side indicating mechanical allodynia. The early administration of the PAFr antagonist (2.4 nmol/d) attenuated the mechanical allodynia significantly compared to the vehicle-treated group (Figure 4A). A low dose of the PAFr antagonist (0.24 nmol/d) partially prevented the mechanical allodynia induced by peripheral nerve injury (Figure 4A). The delayed administration of the PAFr antagonist from 6 days after SNI failed to suppress pain behaviors (Figure 4C). On the contralateral side to the nerve injury, both the early and delayed administration of PAFr antagonist did not have any effect on the withdrawal threshold (Figure 4B, D). These findings indicated that PAF has a role in the development of neuropathic pain following peripheral nerve injury.

### Intrathecal administration of PAF receptor antagonist had no effects on the microglia activation and activation of p38 MAPK activation

**Figure 5:**
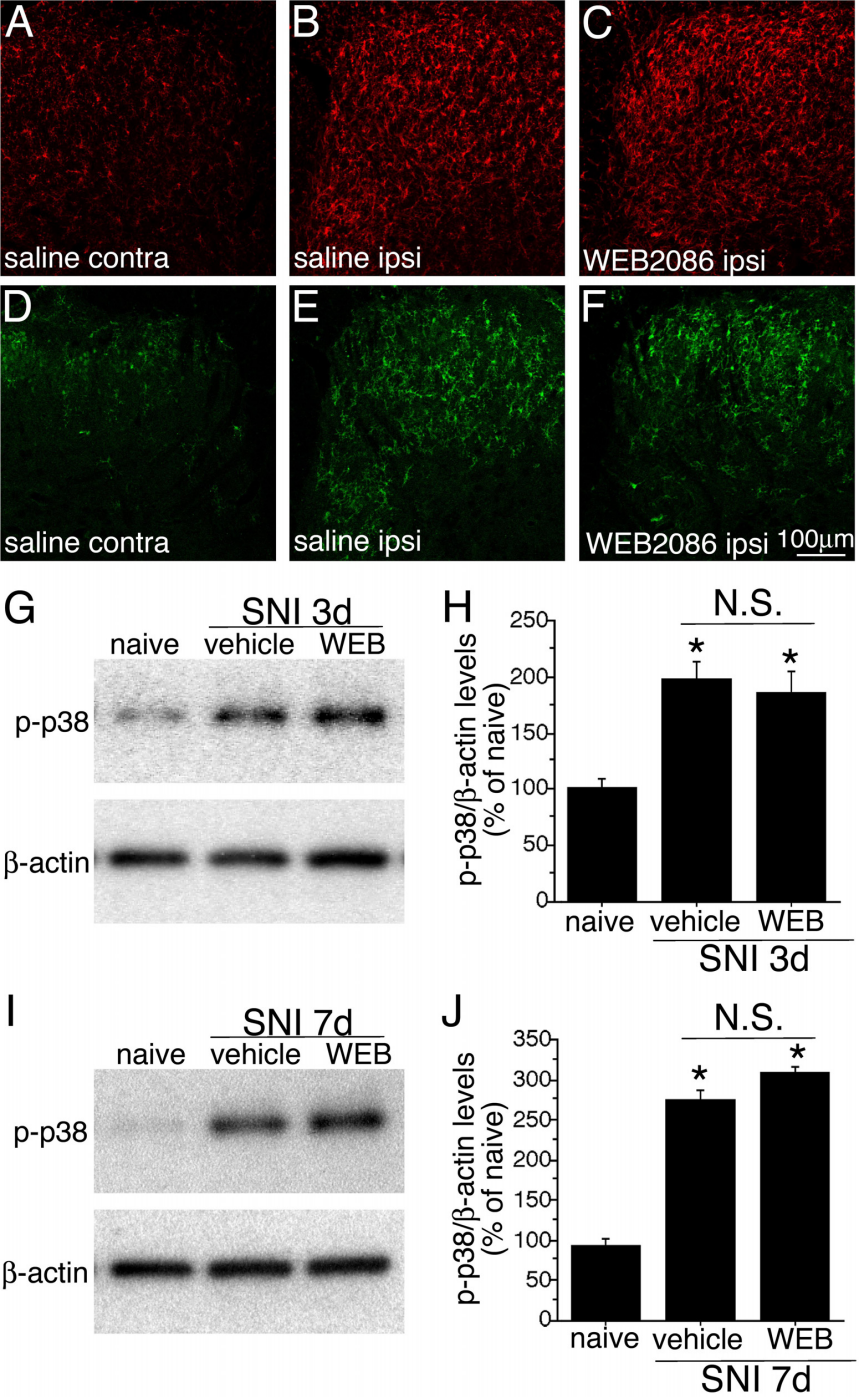
**Intrathecal administration of PAF receptor antagonist (WEB2086) did not affect on the microglia activation and phosphorylation of p38 MAPK following peripheral nerve injury**. (A-C) Immunohistochemistry of Iba1 in the spinal cord of rats that received chronic administration of saline (A, B) and WEB2086 (C) in the contralateral (A) and ipsilateral side (B, C) of dorsal horn at 3 days after SNI surgery. (D-F) Immunohistochemistry of p-p38 in the spinal cord of SNI model rats with saline (D, E) and WEB2086 (F) administration in the contralateral (D) and ipsilateral side (E, F) of dorsal horn at 3 days after SNI surgery. (G-J) Western blot analysis of p-p38 MAPK in the spinal cord after peripheral nerve injury. (G, H) Immunoreactive membrane panels show p-p38 MAPK in the L4, 5 spinal cords at 3 days (G) and 7 days after injury (H). (I, J) Graph shows the protein levels of p-p38 expressed as percentages of the protein level in the normal control spinal cord (mean ± SEM; each time points n = 4,). * indicates *p *< 0.05 (ANOVA) compared to naïve control.

In order to examine the role of PAFr in microglia, we investigated whether intrathecal PAFr antagonist could attenuate microglia activation and p38 MAPK expression in the spinal cord after nerve injury (Figure 5). Immunostaining of Iba1 showed the activation of microglia in the ipsilateral spinal cord to the injury (Figure 5A, B). In the same series of the spinal cord sections, we observed the injury-induced phosphorylation of p38 MAPK (Figure 5D, E). Intrathecal chronic administration of WEB2086 (2.4 nmol/d) from 1 to 3 days after nerve injury did not attenuate the number and intensity of the staining of Iba1 and phosphorylation of p38 MAPK in the spinal cord after nerve injury (Figure 5C, F). Activation of p38 MAPK was examined by Western blot technique (Figure 5G-J). The chronic intrathecal administration of WEB2086 (2.4 nmol/d) from 1 to 3 days (Figure 5G, I) and 1 to 7 days (Figure 5H, J) did not suppress the phosphorylation of p38 MAPK in the spinal cord.

## Discussion

The present study demonstrated the following new findings: (1) Peripheral nerve injury induces mRNA for LPCAT2 but not LPCAT1. LPCAT1 mRNA was localized in spinal cord neurons. LPCAT2 mRNA was constitutively expressed in a small subset of neurons and the increase of mRNA expression of LPCAT2 occurred in spinal microglia, shown by double-labeling analysis of ISHH with IHC. (2) The PAFr is dramatically increased in the spinal cord after nerve injury. The double labeling analysis reveals that PAFr in the dorsal and ventral horn was predominantly colocalized with microglia. (3) Continuous intrathecal injection of a PAFr antagonist significantly inhibits the decrease in withdrawal threshold to mechanical stimuli after peripheral nerve injury. (4) The delayed administration of this antagonist did not attenuate pain behaviors following SNI surgery. Our findings indicate that PAF is produced by microglia and neurons and received by microglia following peripheral nerve injury. Therefore, we postulate that PAF signaling *via *the PAFr among microglia and neurons may be involved in the development of mechanical allodynia after peripheral nerve injury.

PAF, an alkyl-phospholipid, was identified as a platelet-aggregating factor derived from eosinophils [26] and its receptor was cloned by Honda et al. [15]. Accumulating evidence indicates that lipid mediators, such as lysophosphatidic acid (LPA), prostaglandins (PGs) and leukotrienes (LTs) play an important role in neuropathic pain following peripheral nerve injury [9,27,28]. PAF have also been considered an important molecule involved in pathological conditions in the nervous system, such as ischemia-reperfusion injury, spinal cord injury and inflammatory pain [17,18,20]. Furthermore, using PAF receptor-knockout mice, Tsuda et al. reported that PAFr signaling may be involved in producing persistent pain through the mitogen-activating protein kinase (MAPK) in primary afferent neurons after peripheral nerve injury [29]. Intrathecal injection of PAF induced pronounced tactile allodynia, thus suggesting that PAF in the spinal cord may be a mediator of neuropathic pain [24,25]. One important question is which cells produce the PAF and which cells are receptive to PAF in the spinal cord after peripheral nerve injury. There are few reports indicating the localization of PAF synthases and PAFr *in vitro *and *in vivo*. It has reported that LPCAT2 is expressed in primary cultured murine microglia and astrocytes [30] but not neurons derived from brain, and PAFr was found to be expressed in microglia and neurons in the rat hippocampus [31]. In the present study, we demonstrated that LPCAT2 mRNA was expressed in microglia and neurons (Figure 2D, F), and PAFr mRNA induced by peripheral nerve injury was exclusively co-localized with microglia in the spinal cord (Figure 3F). This discrepancy may be due to the difference of the methods (*in vitro *versus *in vivo*) or the regions (hippocampus versus spinal cord).

Microglia, which are considered the macrophages of the central nervous system, are bone marrow-derived haematopoietic cells that infiltrate the central nervous system during embryonic development. Microglia in the spinal cord increase and become activated in the spinal dorsal horn following peripheral nerve injury [32] and activated microglia are known to produce several neurotrophic factors and proinflammatory cytokines, such as brain-derived neurotrophic factor (BDNF), IL-1ß, IL-6 and TNF-alpha, and are involved in the pain hypersensitivity [33-36]. In this study, we showed that PAFr was increased after nerve injury and was predominantly located in microglia in the spinal cord. Recently, it has reported that macrophages around the DRG express PAFr and produce TNF-alpha and IL-1ß after peripheral nerve injury [23]. Our results in the context of previous findings suggest that PAFr is a trigger for the production of proinflammatory cytokines.

Another important question is which intracellular signaling pathway is activated by PAF *via *the PAFr after nerve injury. Previous papers have shown that PAFr-mediated intracellular signal transduction and PAFr may couple with Gi or Gq proteins depending on cell type [10]. It has been demonstrated that PAF-evoked p38 mitogen-activated protein kinase (MAPK) activation is observed in CHO cells expressing PAFr [37]. A lot of reports have defined that nerve injury activates the p38 MAPK cascade in microglia and the activation of p38 MAPK in the spinal microglia contributes to the generation of neuropathic pain [8,38,39]. However, we showed here that PAFr inhibition did not affect on the microglia activation and p38 MAPK phosphorylation in the doses that could suppress neuropathic pain behavior (Figure 5). Taken together with the above-mentioned reports and present study, it may suggest that PAFr activates the signaling cascades that is independent of p38 MAPK and involved in the production of proinflammatory cytokines after peripheral nerve injury.

In the present study, findings that SNI surgery induced upregulation of LPCAT2 and PAFr in spinal microglia and intrathecal injection of PAFr antagonist (WEB2086) significantly suppressed the development of mechanical allodynia suggested an intraspinal role of PAF in neuropathic pain. Because previous studies reported that PAFr is increased in macrophages around DRG in the peripheral nervous system and is involved in hyperalgesia in neuropathic pain condition [23], we could not rule out the possibility that intrathecal injection of PAFr antagonist inhibited the activation of PAFr expressed in macrophages around the DRG. Therefore, we considered that both PAFr expressed by microglia in spinal cord and by macrophages around DRG might be important for neuropathic pain. The delayed administration of PAFr antagonist fail to suppress neuropathic pain related behaviors, suggesting that once PAFr were activated, the downstream signals may turn on the irreversible activation and facilitate the production of proinflammatory cytokines. Our finding indicated that PAF had positive effects on pain behaviors for limited periods after nerve injury as well as other molecules synthesized by activated microglia.

On the other hand, we detected that LPCAT1 and 2 mRNA were constitutively expressed in a subset of spinal neurons. LPCAT1 and 2 have lysophosphatidylcholine acyltransferase activity, which is involved in remodeling of the cell membrane [14,40]. LPCAT1 and 2 in the spinal cord neurons of naive rats, therefore, may constitutively catalyze the cell membrane in the remodeling pathway and also can convert lyso-PAF to PAF. And it has been reported that cPLA2 and LPCAT2 were activated by the increase of intracellular Ca2+ concentration, but not LPCAT1 [14,41]. It is believed that not only in primary afferent neurons, but also in spinal neurons, Ca^2+ ^concentration is up-regulated after peripheral nerve injury [42,43]. After SNI surgery, therefore, we presume that the increase of PAF production could be produced by spinal neurons *via *activation of LPCAT2. The PAF/PAFr signaling may involve neuron/glia communication after nerve injury.

## Conclusions

In summary, the present study indicates that PAF is produced by spinal microglia and neurons, and is received by microglia after peripheral nerve injury. We propose that PAF/PAFr signaling in the spinal cord may act in an autocrine or paracrine manner among the activated microglia and neurons, thus increasing mechanical hypersensitivity.

## Methods

### Animal procedures

Male Sprague Dawley rats weighing 200-250 g were anesthetized with sodium pentobarbital (50 mg/kg, i.p.) and received spared nerve injury (SNI) [44]. The wounds were closed, and the rats were allowed time to recover. At several time points (0, 1, 3, 7 and 14 d) after the SNI, groups of rats were processed for histological analysis (n = 4 at each time point). Every effort was made to minimize animal suffering and reduce the number of animals used. All animal experimental procedures were approved by the Hyogo College of Medicine Committee on Animal Research (#A11-051) and were performed in accordance with the National Institutes of Health guidelines on animal care.

### Reverse transcription-polymerase chain reaction (RT-PCR) and *in situ *hybridization histochemistry (ISHH)

The rats were killed by decapitation under deep ether anesthesia. The ipsilateral of spinal cords (L4-L5) were removed and rapidly frozen with powdered dry ice and stored at -80°C until use. Extraction of total RNA was done using a single step extraction method with ISOGEN (Nippon Gene, Tokyo, Japan) as described in a previous paper [45]. PCR primers for PAF synthases, PAF receptor and glyceraldehyde 3-phosphate dehydrogenase (GAPDH) cDNA were designed as follows:

LPCAT1 (accession number NM_001100735) primers, sense 5'-CCATCCGGCTCCTGTTTGCT-3' and antisense 5'-CGCCCGTCGCTTGATCTCTT-3';

LysoPAFAT/LPCAT2 (accession number XM_001064713) primers, sense 5'-CCTCGCCAGGCGTCCTTCTT-3' and antisense 5'-GAACATGGCACGACCCAGGA-3';

PAF receptor (accession number U04740) primers, sense 5'-GGCTCCTTCCGTGTGGATTC-3' and antisense 5'-GCAGCGGGTGATGTTACCTG-3';

GAPDH (accession number M17701) primers, sense 5'- CCAGGGCTGCCTTCTCTTGT -3' and antisense 5'- CCAGCCTTCTCCATGGTGGT -3'.

The resulting PCR products were used to generate the cRNA probes for ISHH. The rats were killed by decapitation under deep ether anesthesia. The bilateral L4-L5 spinal cord were dissected out, rapidly frozen in powdered dry ice, and cut on a cryostat at a 10 μm thickness. The protocol for ISHH was base on a publish method [46]. Using the enzyme-digested clones, ^35^S UTP-labeled antisense and sense cRNA probes were synthesized. The ^35^S-labeled probes in hybridization buffer were placed on the section, and then incubated at 55°C overnight. Sections were then washed and treated with 1 μg/ml RNase A. Subsequently, sections were dehydrated and air-dried. After the hybridization reaction, the slides were coated with NTB emulsion (Kodak, Rochester, NY, USA) and exposed for 5-6 weeks. Once developed in D-19 (Kodak), the sections were stained with hematoxylin-eosin and coverslipped.

### Double-labeling analysis of *in situ *hybridization with immunohistochemistry

To examine the distribution of mRNAs for PAF synthases and its receptor in neurons versus glial cells, we used a combined ISHH with immunohistochemistry (IHC). The frozen spinal cord sections were processed for IHC using the ABC method [47]. The following antibodies for double-labeling analysis were used: rabbit anti-ionized calcium-binding adapter molecule1 (Iba1) polyclonal antiserum (1:100; Wako Chemicals, Tokyo, Japan), mouse anti-neuronal specific nuclear protein (NeuN) monoclonal antiserum (1:2000; Chemicon, Temecula, CA), and rabbit anti-glial fibrillary acidic protein (GFAP) polyclonal antiserum (1:2000; DakoCytomation, Glostrup, Denmark). Several markers were visualized as brown signals by 0.05% 3, 3-diaminobenzidine tetrahydrochloride (DAB; Sigma) containing 0.01% hydrogen peroxidase without nickel sulfate. After IHC, these sections were immediately processed for ISHH. A detailed description of the treatment of sections and methods of double labeling with IHC and ISHH were described previously [48].

### Fluorescence immunohistochemistry

IHC was performed as described before [49]. The tissue was frozen in powdered dry ice, cut on a cryostat at a 25 μm thickness. The following antibodies were used for IHC: rabbit anti phosphorylated p38 polyclonal antiserum (1: 1000, Cell signaling MA, USA), goat anti Iba1 polyclonal antiserum (1:500, Abcam, Cambridge, MA). In brief, spinal cord sections were incubated with a primary antibody over night at 4°C and followed by secondary antibodies; anti rabbit Alexa Fluor 488 IgG (1:1, 000; Invitrogen, San Diego, CA), anti goat Alexa Fluor 594 IgG after incubation with respective primary antibodies.

### Western blot

The spinal samples preparation and Western blotting were performed as described before [49]. Membranes were incubated with Blocking One P (Nakarai, Kyoto, Japan) in Tris buffer containing Tween 20 (TBST) (10 mM Tris-HCl, pH 8.0, 150 mM NaCl, and 0.2% Tween 20) for 20 min at room temperature and incubated with the polyclonal primary antibody for phosphorylated p38 (1:2000; Cell signaling) at 4°C overnight. Membranes were then washed twice with TBST and probed with goat anti-rabbit IgG conjugated with alkaliphosphatase (1:2000, Chemicon) at room temperature for 2 h and visualized by chemiluminescence using CDP-star ready-to-use reagent (Roche, Indianapolis, USA). The loading and blotting of the amount of protein was verified by reprobing the membrane with anti-β-actin antiserum (1:2000; Sigma). Films were scanned and quantified using NIH Image, version 1.61 and normalized against a loading control (beta-actin). Data are expressed as mean ± SEM. Differences in changes of values over time of each group were tested using one-way ANOVA, followed by individual post hoc comparisons (Fisher's exact test). A difference was accepted as significant if *p *< 0.05.

### Drug treatments

Two or six days after SNI surgery, the L5 vertebra was laminectomized under adequate anesthesia with sodium pentobarbital, and a soft tube (Silascon, Kaneka Medix Company, Osaka, Japan; outer diameter, 0.64 mm) filled with 5 μl of saline was inserted into the subarachnoid space for an ~0.5 cm length (tube were pointed caudally). Mini-osmotic pumps (model 2001; 7 days pump, 1 μl/h, Alzet Corporation, Palo Alto, CA) filled with saline or PAFr antagonist (WEB2086) [4-[3-[4[(2-Chlorophenyl)-9-methyl-6H-thieno[3, 2-f][1,2,4]triazolo[4, 3-a]diazepin-2-yl]-1-oxopropyl]morpholine]] (Tocris Bioscience, MO, USA) were connected to the tube. Then, the pump was laid under the skin and the incision was closed. The concentrations of WEB2086 was 0.24 nmol/day and 2.4 nmol/day diluted in 10% dimethyl sulfoxide (DMSO) (n = 6-7, for behavior test).

### Behavioral tests

All SNI rats were tested for mechanical allodynia on the plantar surface of the hindpaw 1 day before surgery and 1, 3, 5, 7, 9, 11 and 14 days after surgery. Mechanical allodynia was assessed with a dynamic plantar anesthesiometer (Ugo Basile, Comerio, Italy), which has an automated von Frey-type filament (0.5 mm diameter) [50,51]. The detailed method of mechanical sensitivity measurement in rat hindpaw was described previously [49].

### Statistics

Data are expressed as mean ± SEM. Differences in changes of values over time of each group were tested using one-way ANOVA, followed by individual *post hoc *comparisons (Fisher's exact test) or pair-wise comparisons (*t *test) to assess differences of values between naive versus each time point of the SNI groups. A difference was accepted as significant if *p *< 0.05.

## Competing interests

The authors declare that they have no competing interests.

## Authors' contributions

MO carried out the histological studies, performed the statistical analysis, and participated in drafting the manuscript. HY, KK, HK helped the histological studies and performed the behavioral pharmacology experiments. YD and KN conceived of the project, designed and coordinated the studies, and drafted and edited the manuscript. All authors contributed to data interpretation, have read and approved the final manuscript.
